# The Nutraceuticals as Modern Key to Achieve Erythrocyte Oxidative Stress Fighting in Osteoarthritis

**DOI:** 10.3390/cimb44080240

**Published:** 2022-08-05

**Authors:** Alessia Mariano, Irene Bigioni, Francesco Misiti, Luigi Fattorini, Anna Scotto d’Abusco, Angelo Rodio

**Affiliations:** 1 Department of Biochemical Sciences, Sapienza University of Rome, 00185 Rome, Italy; 2 Department of Human Sciences, Society and Health, University of Cassino and Southern Lazio, 03043 Cassino, Italy; 3 Department of Physiology and Pharmacology, Sapienza University of Rome, 00185 Rome, Italy

**Keywords:** oxidative stress, erythrocytes, osteoarthritis, nutraceuticals, *Harpagophytum procumbens*, *Boswellia serrata*, *Curcuma longa*

## Abstract

Osteoarthritis (OA), the most common joint disease, shows an increasing prevalence in the aging population in industrialized countries. OA is characterized by low-grade chronic inflammation, which causes degeneration of all joint tissues, such as articular cartilage, subchondral bone, and synovial membrane, leading to pain and loss of functionality. Erythrocytes, the most abundant blood cells, have as their primary function oxygen transport, which induces reactive oxygen species (ROS) production. For this reason, the erythrocytes have several mechanisms to counteract ROS injuries, which cause damage to lipids and proteins of the cell membrane. Oxidative stress and inflammation are highly correlated and are both causes of joint disorders. In the synovial fluid and blood of osteoarthritis patients, erythrocyte antioxidant enzyme expression is decreased. To date, OA is a non-curable disease, treated mainly with non-steroidal anti-inflammatory drugs and corticosteroids for a prolonged period of time, which cause several side effects; thus, the search for natural remedies with anti-inflammatory and antioxidant activities is always ongoing. In this review, we analyze several manuscripts describing the effect of traditional remedies, such as *Harpagophytum procumbens, Curcumin longa*, and *Boswellia serrata* extracts, in the treatments of OA for their anti-inflammatory, analgesic, and antioxidant activity. The effects of such remedies have been studied both in in vitro and in vivo models, considering both joint cells and erythrocytes.

## 1. Introduction

Osteoarthritis (OA) is a chronic, degenerative, and inflammatory disease that affects articular joints, causing pain and functional limitation in patients. Knee, hip, and the small articular joints in the hand are mainly involved [1]. For several years, OA disease was called Arthrosis and considered only a pathology linked to the aging process. Recently, scientific studies demonstrated the involvement of inflammation in OA pathogenesis, which now is officially recognized as Osteoarthritis. The desinence “itis” indicates quantitatively variable inflammation, which is present in each phase of the disease [2]. OA affects more than 20% of the general population [3] and is significantly increased among retired elite athletes, with prevalence rates as high as 95% [4]. The incidence of OA increases with age, and women have higher rates than men, especially after age 50. A leveling off or decrease in incidence occurs at all joint sites around the age of 80 [5]. The prevalence and incidence of OA across studies vary greatly. It depends on the population sampled (primary versus tertiary care) and risk factors, such as age, sex, obesity, and geographical region [1].

Nowadays, there is no cure for OA disease; all the therapies are aimed to reduce pain and inflammation and improve the patient quality of life. Drugs mainly administered are NSAIDs, corticosteroids, and analgesics to counteract symptoms. Considering that OA is a chronic disease, severe side effects can be attributed to prolonged drug administration. For this reason, further studies have focused their attention on alternative treatments. Considering the OA cartilage damage, chondroprotective agents are being analyzed. Glucosamine and its derivative [6,7], chondroitin sulfate [8], or hyaluronic acid [9] are studied as valid therapeutic options. Traditional remedies, among them *Harpagophytum procumbens, Curcumin longa, Boswellia serrata*, and others co-adjuvate chondroprotectors for their anti-inflammatory and analgesic activity. These effects can be attributed to the high content of flavanols, flavonoids, triterpenes, and volatile molecules, bioactive compounds showing an antioxidant activity [10,11,12,13].

In this narrative review, we considered recent progress in OA treatment using traditional remedies, such as *Harpagophytum procumbens, Curcumin longa*, and *Boswellia serrata* extracts. Mainly the anti-inflammatory, analgesic, and antioxidant activity of these extracts have been taken into consideration. Manuscripts describing the effects of such remedies both in in vitro and in vivo models, considering both joint cells and erythrocytes have been analyzed.

## 2. Oxidative Stress and Inflammation

Oxidative stress is defined as an imbalance between the production of reactive oxygen species (ROS) and their elimination by protective mechanisms. In oxidative stress, there is an over-production of ROS and oxidative species, responsible for the overexpression of various transcription factors and several genes involved in inflammatory pathways [14,15]. The inflammation triggered by oxidative stress is typical of chronic diseases such as OA: many authors documented a link between oxidative stress and OA inflammation [16,17,18]. In OA, ROS are among the most involved agents in modifying proteins and lipids, damaging DNA, and other adverse effects on joint cells [19,20,21]. Erythrocytes (RBCs) belong to those cell types in which a redox imbalance can occur. Fatty acids in the membranes elevated oxygen levels, and the presence of hemoglobin makes RBCs a natural target for free radicals [22]. However, RBCs are endowed with extraordinarily efficient enzymatic and non-enzymatic antioxidant defense systems that act as ROS scavengers to limit their cellular damage [23].

## 3. RBCs and Oxidative Stress

Erythrocytes (RBCs) are the most abundant blood cell (4.2–6.1 × 10^9^/mL in humans), higher in men than in women. Consequently, RBCs comprise a large percentage of the blood volume [24]. Their primary function is oxygen (O_2_) and, to some extent, carbon dioxide (CO_2_) transport to and from tissues, by virtue of binding of the gases to hemoglobin (Hb) [25]. RBCs are cells derived from hematopoietic stem cells and have an average life span of 120 days ± 20–30 days, in which they are exposed to large stressful situations. Considering that they have to cross through capillaries, smaller than cells, the integrity of the RBCs membrane is constantly affected [26].

For these reasons, the RBCs membrane has unique biophysical features which make it highly elastic and deformable. RBCs membrane has a two-dimensional structure, composed of a cytoskeleton and a lipid bilayer. The lipid bilayer includes various types of phospholipids, sphingolipids, cholesterol, and integral membrane proteins, such as band-3 and glycophorin [27,28]. Considering that RBCs are responsible for oxygen transport in the blood (Reaction 1), their high amount and related ROS production are one of the main causes of lipid and protein membrane oxidative damage. Endogenous ROS are produced during spontaneous oxidation when the ferrous ion (Fe^2+^) in the heme group is converted to a ferric state (Fe^3+^) (Reaction 2). Reactions that occur during Hb auto-oxidation are [29]:

Hb(Fe^2+^) + O_2_ ⇄ Hb(Fe^2+^)O_2_ (1)

Hb(Fe^2+^)O_2_ ⇄ Hb(Fe^3+^)O_2_ (2)

Hb(Fe^3+^)O_2_ → Hb(Fe^3+^) + O_2_^•−^ (3)

Excess ROS production irreversibly damages lipids and proteins of the RBCs membrane because mature RBCs have no nucleus and other cell organelles, so they have no capacity to repair the damaged components [30]. Due to the presence of polyunsaturated fatty acids (PUFA), the cell membrane is susceptible to free radicals. Indeed, long-chain polyunsaturated fatty acids, including docosahexaenoic acid (DHA, C22:6n−3) and arachidonic acid (AA, C20:4n−6), contain multiple double bonds in between which lie methylene bridges (-CH_2_-) that possess hydrogen atoms particularly reactive with ROS [30,31]. PUFAs oxidative damage leads to lipid peroxides formation and to reactive carbonyl electrophiles releases, such as malondialdehyde (MDA) and 4-hydroxynonenal (HNE). These electrophile compounds cause harm by reacting with cellular components such as proteins and nucleic acids [32,33]. Proteins are the main target of oxidative damage due to their rapid reaction rate with both lipid peroxidation products and oxygen radicals. ROS are responsible for the oxidation of the side chains of amino acid residues, the formation of protein-protein crossbonds, and the oxidation of the protein backbone resulting in protein fragmentation, morphologic, biochemical, and metabolic changes in RBCs [34]. There are proteins involved in interactions between RBCs and other blood cells and endothelial cells; other ones are receptors implicated in signal transduction or proteins with transport function [35]. In particular, due to their high nucleophilicity, thiols groups in erythrocyte proteins are very vulnerable to oxidative stress induced by ROS, and their oxidation is responsible for alterations in protein structure and function [36]. In the presence of ROS, sulfhydryl protein residues may undergo reversible oxidation: sulfhydryl bonds are broken, and disulfides are formed [37]. However, thiols not only represent one of the main targets of ROS, but also a versatile and robust defense system against oxidative stress [36]. Damage to membrane proteins is, presumably responsible for the impaired cellular deformability associated with oxidative stress [38]. Decreased deformability of RBCs can also cause impaired oxygen delivery, which contributes to the pathology of a number of diseases, such as diabetes, sickle cell anemia, malaria, and some neurodegenerative diseases [39,40,41,42].

RBCs act against oxidative damage both with an enzymatic antioxidant system and a non-enzymatic one. Superoxide dismutase, glutathione peroxidase, glutathione reductase, and catalase are the most active antioxidant enzymes [43]. Superoxide dismutase enzyme (SOD) is a copper-containing enzyme that converts two molecules of superoxide into oxygen and hydrogen peroxide (Reaction 4) [44].

O_2_^•−^ + O_2_^•−^ + 2H^+^ → O_2_ + H_2_O_2_ (4)

Hydrogen peroxide is subsequently detoxified to oxygen and water by glutathione peroxidase or catalase. Glutathione peroxidase (GPX) is a selenoprotein that catalyzes the reduction of H_2_O_2_ into two molecules of water (Reaction 5) [44]. The oxidized glutathione (GSSG) is transformed into its reduced form (GSH) by glutathione reductase (GR), which plays a vital role in protecting RBCs against oxidative damage [45,[46]].

H_2_O_2_ + 2 GSH → 2H_2_O + GSSG (5)

Catalase enzyme represents an alternative mechanism to protect RBCs from oxidative damage induced by hydrogen peroxide, transforming the latter into water and molecular oxygen (Reaction 6) [46].

2H_2_O_2_ → 2H_2_O + O_2_ (6)

Moreover, RBCs are well equipped with a non-enzymatic antioxidant system that includes GSH, vitamin C, vitamin E, and NADH/NADPH cofactors [47]. GSH is a tripeptide constituted by the three amino acids L-glutamate, L-cysteine, and L-glycine, that through cysteine -SH function keeps thiol groups of Hb, enzymes, and membrane proteins in the reduced form [48]. Vitamins C and E are recognized as free radical scavengers with a synergistic effect and are involved in various antioxidant mechanisms, including the protection against lipid peroxidation. Vitamin C, as a water-soluble molecule, is able to exert its antioxidant activity in the cytoplasm, while the lipid-soluble vitamin E is more active in cell membranes [49]. Dehydroascorbate, vitamin C anionic form at physiological pH, has a protective role against lipids peroxidation of erythrocyte membrane and in tocopherols oxidation. When vitamin E is protecting lipid from peroxidation, donates an electron to a lipid peroxyl radical, converting itself into a radical form stabilized by resonance. The dehydroascorbate anion reduces tocopheroxy radicals, restoring the antioxidant properties of vitamin E [50]. On the other hand, NADH/NADPH cofactors are considered essential for the catalytic activity of both major H_2_O_2_ catabolizing pathways, such as catalase and superoxide dismutase, and glutathione peroxidase enzyme. This couple of cofactor is indispensable for RBCs redox homeostasis and energy metabolism, an imbalance of the redox state of these molecules is implicated in various pathological conditions [51].

## 4. Osteoarthritis and RBCs Oxidative Stress

**Figure 1:**
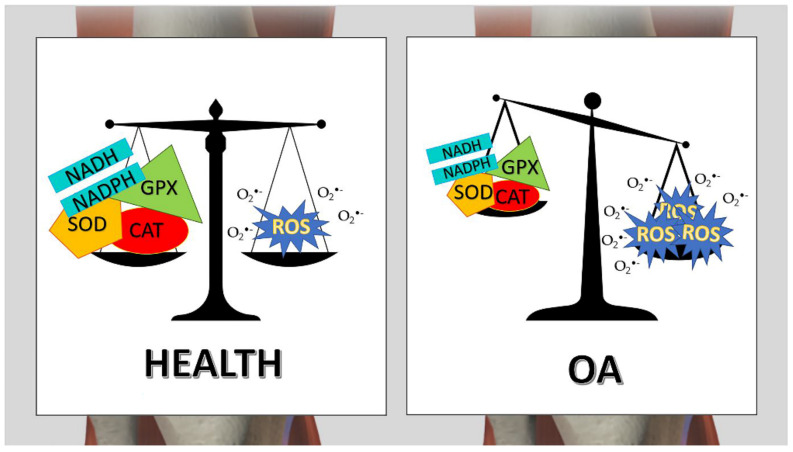
Oxidative stress in OA disease. The balance between anti -ROS molecules and ROS, production is reported both in healthy and OA joints.

Oxidative stress and inflammation are highly correlated and are both causes of joint disorders. Several studies demonstrated that erythrocyte antioxidant enzyme expression is decreased in synovial fluid of osteoarthritis and rheumatic patients [52,53,54]. Activities of some erythrocyte antioxidant enzymes, such as SOD, GPX, and catalase in osteoarthritis (OA) and rheumatoid arthritis (RA), are reduced with respect to physiological conditions (Figure 1). If ROS are not scavenged, these species may lead to damage to lipid, protein, and DNA. It was observed an increase in RBCs lipid peroxide products [55]. The most elevated compound is malondialdehyde (MDA), considered a marker of cellular oxidative damage in pathological conditions, including OA [56]. Furthermore, a decrease in non-enzymatic defensive mechanism has been detected by Surapaneni KM et al. [57], and significantly lower levels of Vitamin E and Vitamin C were found in OA patients with respect to healthy ones.

## 5. Osteoarthritis and Antioxidant Treatment

As previously described, osteoarthritis (OA) is a chronic and degenerative joint disease that requires prolonged treatments to counteract the symptoms. NSAIDs and corticosteroids are the most common pharmacological remedies. However, their chronic administration is associated with dangerous side effects and arthroplasty surgery often is inevitable. For these reasons, in order to slow down OA progression, diminish the drug assumption period and postpone the surgery, nutraceuticals can be considered a good alternative during the early stage of the disease.

The term nutraceutical is composed of ‘nutrition’, as dietary supplement, and ‘pharmaceutical’, according to its physiological benefits. However, the term nutraceuticals is not recognized by the US Food and Drug Administration (FDA), which uses the term ‘dietary supplements’ [58]. Nutraceutical is a food providing health improvements in addition to their nutritional value; it contains bioactive compounds with a pharmacological effect [59]. Many bioactive compounds are known, but new molecules are constantly isolated and identified. They can be classified into hydrophilic and hydrophobic compounds, among them, polyphenols and terpenoids are the most representative [59,60].

Polyphenols are common nutrients mainly derived from fruits, vegetables, tea, and the traditional medicinal herb [61]. They are characterized by bitterness, astringent color, and odor and protect plants against oxidative processes. In vivo polyphenols show anti-inflammatory and anti-nociceptive effects and are involved in the activation of Nrf2, a transcription factor fundamental in cellular protection against oxidative stress [14,62]. The classification of polyphenols includes flavonoids (60%), phenolic acids (30%), and other polyphenols attached to at least one aromatic ring with one or more hydroxyl functional groups [61]. The antioxidant potential of polyphenols depends on the number and position of the hydroxyl groups, which defines their scavenging potential of ROS, a high number of hydroxyl groups shows a higher antioxidant capacity [63]. Terpenes are volatile compounds produced by plants as a defense against bacteria, fungus, and insects [64]. Their chemical nomenclature is based on the number of isoprene units that they contain, this is the reason why they are classified as sesquiterpenes, monoterpenes, diterpenes, triterpenes, tetraterpenes, and polyterpenes [65]. In vitro studies showed that some terpenes have significant antioxidant effects and chondroprotective activity in a cellular model of human chondrocytes [66]. For this reason, this effect results in slowing down the destruction of cartilage and OA progression [67].

In plants, these molecules serve as antioxidant compounds and as protection against high temperatures, drought, or excessive light intensity [68]. As well as humans, also plants have protective mechanisms against oxidative stress induced by singlet oxygen and by ROS produced during chlorophyllin photosynthesis. In human inflammatory diseases, such as OA, the erythrocyte enzymatic and non-enzymatic antioxidant systems are altered. For this reason, the assumption of nutraceuticals, which contain a broad spectrum of antioxidants, such as polyphenolic compounds and terpenoids, can protect the organism against oxidative damage [63,69].

This review aims to discuss the involvement of nutraceuticals typically used in OA treatment against oxidative stress with the support of previous scientific results. *Harpagophytum procumbens*, *Boswellia serrata* and *Curcuma longa* will be the object of this paper.

### 5.1. Harpagophytum Procumbens

*Harpagophytum procumbens*, also known as devil’s claw, is used in Europe during the last decades in traditional medicine for joint disease. Devil’s claw is native to the southern part of the African continent and may be found in Namibia, Botswana, South Africa, Angola, Zambia, and Zimbabwe [70,71]. Several studies indicate that Devil’s Claw root extract is an effective treatment in OA because of its anti-inflammatory and anti-nociceptive activity. Katarina Hostanska et al. have observed *Harpagophytum procumbens* extract (HPE) in vitro effect on the THP-1 cell line, a leukemic cell line, demonstrating a decrease in pro-inflammatory cytokines (IL-6, IL-8, and TNF-α) levels following lipopolysaccharide stimulus [72]. Additional evidence of the anti-inflammatory and chondroprotective effect is shown in Schulze-Tanzil et al. experiments. HPE inhibited metalloproteases (MMPs) released by chondrocytes, consequently preventing cartilage damage [73]. Moreover, the agonism of some HPE components has been shown on endocannabinoid (CB) receptors, mainly involved in anti-nociceptive transmission. Several studies have highlighted the up-regulation of CB2 following HPE treatment in vitro [74,75]; while in Farpour et al. in vivo study, pain reduction following HPE administration was demonstrated [76]. Its in vitro and in vivo activity is due to the major chemical constituents such as iridoid glycosides (primarily harpagoside, harpagide, and procumbide), triterpenoids, phytosterols (primarily β-sitosterol) and flavonoids such as luteolin and kaempferol [77]. Harpagoside, harpagide, and procumbide, found in the tubers of the plant, appear to be the most therapeutically important constituents. Whole-plant extracts appear to have a better therapeutic effect than those prepared from isolated parts [71,75].

As previously described an inflammatory environment leads to an increase in ROS production and an alteration of oxidant/antioxidant balance. The HPE antioxidant activity is mainly attributed to flavonoid and phenol compounds present in the devil’s claw. For this reason, several studies have focused their attention on the antioxidant effect of HPE, evaluating superoxide dismutase, catalase, glutathione peroxidase enzyme activity, ROS decreased, and lipid peroxidation inhibition. The antioxidant profile of HPE was mainly studied in vivo and correlated to neurodegenerative diseases, although oxidative stress is largely involved in all inflammatory pathologies, including OA. Peruru et al. analyzed GSH, SOD, and CAT levels in arsenic-induced oxidative stress rat models [78]. Doses of 200 and 400 mg/kg, p.o of HPE resulted able to restore basal levels of erythrocyte antioxidant enzymes. On the other hand, MDA and NO content was also alleviated compared to the arsenic control group supporting the antioxidant properties of HPE. Its effect on antioxidant profile resulted to be dose-dependent [78]. These results supported data previously obtained by Bhattacharya and Bhattacharya in 1998. They demonstrated an effect in increasing erythrocyte levels of GSH, SOD, and CAT, and also an additional antioxidant activity affecting erythrocyte GPX amount in animals treated for at least 7 days with HPE [79]. More recently, in vitro analysis aimed to test the antioxidant properties of HPE on lipid peroxidation and ROS production. DPPH radical scavenging assay, oxygen radical absorbance capacity (ORAC), and hydroxyl radical averting capacity (HORAC) tests are usually used. HPE or its single components alone resulted able to break the radical chain and scavenge both superoxide and peroxyl radicals, indicating a good antioxidant activity in vitro [12,80,81].

Taken together these results allow us to consider *H. procumbens* as a valid therapeutic remedy against oxidative stress. Its effect on ROS reduction and antioxidant enzyme increase can contribute to the anti-inflammatory activity observed in OA patients.

### 5.2. Boswellia Serrata

The gum resin of *Boswellia serrata* (BS) has been used for centuries in traditional medicine as a remedy for many health problems. B*oswellia serrata* (*Salai/Salai guggul*) is a moderate to large-sized branching tree of the family Burseraceae, that grows in India, Northern Africa, and Middle East mountains. Oleo gum-resin is tapped from the incision made on the trunk of the tree and then solidified in special bamboo baskets [82]. It contains essential oil, mucopolysaccarides, pure resin with monoterpenes, diterpenes, triterpenes, tetracyclic triterpene acids, and pentacyclic triterpene acids, called boswellic acids (BAs). Six major boswellic acids have been isolated: keto-*β*-boswellic acid (KBA), 3-O-acetyl-11-keto-*β*-boswellic acid (AKBA), *α*-boswellic acid (*α*-BA), *β*-boswellic acid (*β*-BA), 3-O-acetyl-*α*-boswellic acid (*α*-ABA), and 3-O-acetyl-*β*-boswellic acid (*β*-ABA) are considered the most important bioactive molecules [82,83]. As a potential anti-inflammatory treatment, the efficacy of *Boswellia serrata* extract (BSE) has been reported in many clinical trials during the last 20 years [84,85,86]. These studies demonstrate that oral supplementation with BSE prevents articular cartilage degradation decreases osteophytes formation, and consequently improves physical mobilization by reducing pain in OA patients compared with placebo control [84,87]. Interestingly, the clinical trial reported by Sontakke et al. indicates that the onset of BSE action is slow but at the same time its effect was persistent even at the end of the treatment [86].

The observed anti-inflammatory and anti-nociceptive effects of BSE in clinical trials have been also demonstrated in several in vitro studies. 5-lypoxigenase (5-LO), which catalyses the synthesis of leukotrienes from arachidonic acid, has been proposed as a specific target for boswellic acids. [82]. Moreover, Boswellic acids and BSE showed their anti-inflammatory effect by reducing the production of inflammatory cytokines, including IL-1β, IL-6, IFNγ, and TNFα that are ultimately directed to cartilage destruction in OA. However, even if the biochemical mechanism of anti-inflammatory action is still the object of studies, recent evidence demonstrated a direct interaction with IκB kinases and the nuclear factor-κB (NF-κB) complex. In vitro experiments performed by Takada et al. showed that AKBA was able to inhibit inducible and constitutive NF-κB activation and IKK activation through Akt suppression, during osteoclastogenesis. This event leads to the reduction of IκBα post-translational modifications and its degradation, p65 phosphorylation, and its nuclear translocation, and finally the decrease in NF-κB-correlated gene expression [88]. On the other hand, to investigate the BSE chondroprotective role Sengupta et al. performed in vitro experiments on human primary chondrocytes and synoviocytes. *Boswellia* products resulted able to stimulate cellular proliferation and the glycosaminoglycans synthesis in chondrocytes, and inhibit MMP-3 production in TNF-α-induced synoviocytes [89]. These data together allow us to consider BSE as a good alternative OA treatment for its potential in recovering articular cartilage damage or protecting from proteolytic degradation due to inflammatory factor release.

These protective effects in OA have been also correlated to BSE antioxidant activity by suppressing ROS levels, lipid peroxidation, and decreasing oxidative DNA damage. Catanzaro et al. studied the effect of BSE against oxidative stress in a chronic inflammatory disease cellular model on the Caco-2 cell line. They demonstrated that BSE and AKBA at the same concentration resulted able to prevent the ROS intracellular production, stimulated by H_2_O_2_ treatment, consequently ameliorating the intestinal damage induced by oxidative stress and inflammation [90]. These results are in line with the data obtained by Avasthi et al. on H_2_O_2_-stimulated human RBCs. BSE treatment generated a significant ROS reduction decreasing LPO production and erythrocyte hemolysis by protecting membrane integrity [91]. Regarding the BSE antioxidant effect on enzymes altered in oxidative stress, an in vivo study was performed by Umar et al., using OA rat models. Joint damage was induced by injection of collagen fragments and then ethanol BSE was administered for 21 days. Treatment with BSE significantly increased GSH, SOD, and CAT levels, boosting the antioxidant defense system [92].

All these results suggest that *Boswellia serrata* could be considered an antiarthritic remedy for joint diseases by controlling inflammatory pathways, reducing oxidative stress, and finally protecting cartilage from degradation.

### 5.3. Curcuma Longa

*Curcuma longa Linn*. is one of the most investigated natural products considered very beneficial in OA prevention and treatment. *Curcuma longa Linn.*, commonly known as “The gold spice” belongs to the family Zingiberaceae, it is an indigenous plant of India, also cultivated in China, Sri Lanka, and other tropical countries. Roots contain non-volatile and volatile bioactive compounds used in traditional medicine [93]. The volatile compounds, such as terpenoids, and flavonoids, are responsible for the *C. longa* aroma, while the non-volatile compounds known as curcuminoids are responsible for their bright yellow color. Among these non-volatile compounds, three curcuminoids, named curcumin, demethoxycurcumin, and bisdemethoxycurcumin, have shown medical properties. Their pharmacological action occurs by different mechanisms. Different studies described the curcumin effect in regulating the release of inflammatory mediators and in modulating signaling pathways [94]. Buhrmann et al. mimicked an osteoarthritic environment creating a multicellular model composed of fibroblasts, chondrocytes with T-lymphocytes, and 3D-alginate. They found out how curcumin suppresses NF-κB activation, by directly inhibiting DNA binding of p65-NF-κB, and stimulates Sox-9 production, a cartilage-specific protein [95]. Considering the close correlation between OA and oxidative stress, Srivastava and colleagues performed a double-blind, randomized, placebo-controlled clinical trial to investigate the in vivo effect of CLE. They correlated the severity of the disease and the hematic levels of IL-1β, ROS, and biomarkers of oxidative stress finding out that four months of CLE treatment improves joint condition by decreasing inflammatory and oxidative mediators [96]. These promising results find support also in in vivo animal studies. It was demonstrated that 21 days of treatment with CLE is responsible for an increase in antioxidant enzyme levels. Hematic SOD, CAT, GPx activity, and NADPH amount were positively affected by CLE in mouse samples with respect to placebo control. The higher activity of these antioxidant enzymes is responsible for ROS release inhibition and finally a reduction in MDA production [97].

The CLE protective activity against oxidative stress has been also the object of various in vitro studies. Singh and colleagues in 2015 evaluated an overactivation of Na^+^/H^+^ exchanger (NHE) and down-activation of Na^+^/K^+^ ATPase (NKA) caused by the oxidative damage of tert-butylhydroperoxide (t-BHP) 30 min treatment on erythrocyte membrane. NHE is a ubiquitous electroneutral, ion exchanger involved in cell volume, cellular growth and differentiation, and cell motility. Similarly, ion-transporter NKA, is a heterodimeric transmembrane ion pump acting as a signal transducer, regulating ionic gradients across the cell membrane and its osmotic equilibrium. It has been observed that curcumin mitigated oxidative stress by reducing the ROS synthesis, increasing GSH concentration, and consequently reversing the NHE overactivity. On the other hand, curcumin increased the NKA activity in RBCs by interacting with some amino acids (Thr, Glu, Val, Arg, Tyr, Gly, Ser, Ile, Phe, Tyr, and Ile) at the active site cavity of the pump [98]. Moreover, recently curcumin has been identified as a potent inducer of heme oxygenase-1 (HO-1), a redox-sensitive inducible protein that provides protection against oxidative stress degrading heme to CO, iron, and biliverdin. In cultured astrocytes, curcumin activated the HO-1 gene via restoring Nrf2 function and causing transient and marked changes in the intracellular GSH/GSSG ratio. In line with this effect, the authors speculated that plant curcuminoids increased HO-1 activity because their thiol groups react with cysteines of different proteins involved in signal-transduction antioxidant pathways [99]. These observed results are common to many in vitro and in vivo studies, on human and on animal models, confirming the antioxidant activity of CLE.

## 6. Conclusions

Osteoarthritis (OA) is a chronic and degenerative joint disease that leads to cartilage degradation involving synovial inflammation and subchondral bone remodeling. Cartilage degradation results from catabolic processes activated by pro-inflammatory mediators such as cytokines, lipid mediators, and reactive oxygen species (ROS). ROS are normally produced within the body in a limited amount and are essential compounds involved in the regulation of processes capable of maintaining cell homeostasis and functions. For these reasons, ROS have two faces: the first is its participation in redox signaling and the second is its role in oxidative stress or injury. A ROS overproduction is responsible for oxidative stress, which can lead to lipid peroxidation, DNA damage, and other adverse effects [100]. Notably, circulating RBCs are very susceptible to oxidative stress due to the high content of polyunsaturated fatty acids in their lipid bilayer, continuous exposure to high oxygen levels, and the auto-oxidation of hemoglobin [101]. Even if these cells are excellently equipped with enzymatic and non-enzymatic antioxidant systems to counteract intracellular oxidative stress, often these defense mechanisms are not enough.

Different studies demonstrated that OA progression is significantly related to oxidative stress and ROS level. Indeed, in the blood of patients with knee OA, increased ROS level, lipid peroxide amount, and decreased antioxidant protection are detected and correlated to inflammation and cartilage degradation. Moreover, erythrocyte antioxidant enzymes, such as SOD, GPX, and catalase in chronic joint diseases are found reduced with respect to physiological conditions. Consequently, if ROS are not counteracted, lipid, protein, and DNA damage occurs. In OA, joint damage is not just limited to articular cartilage, but also affects the subchondral bone and the synovial membrane. Articular cartilage is a uninervate and avascular tissue with unique properties and therefore not directly affected by erythrocyte oxidative stress. Indeed, chondrocytes, the only constitutive cartilage cells, have no detectable mitotic activity and are adapted to live in an environment with a low oxygen supply. Due to oxygen diffusion from neighbouring tissues, the superficial and middle areas of cartilage are partially exposed to ROS [102]. On the contrary, the synovial membrane is a vascular tissue highly sensitive to oxygen radical species produced by RBCs and brought through hematic flow. The increase in ROS concentration, produced by both RBCs and synovial cells, is responsible for various tissue damage. This oxidative stress can lead to the development of synovitis, an inflammatory condition characterized by higher MMPs and other degradative proteases amount, which exacerbates cartilage destruction.

Nutraceutical is a food that contains bioactive compounds with a pharmacological effect that provides health improvements. In last years, nutraceuticals have been largely assumed in addition to traditional drugs as an alternative OA treatment for the early stage of pathology, considering their safety and efficacy. *Harpagophytum procumbens*, *Boswellia serrata*, and *Curcuma longa* are three of the most studied antiarthritic plant extracts and are recognized as valuable remedies against joint diseases. Their administration dates to the days of traditional medicine, but only recently their biochemical mechanisms have been understood. In addition to the anti-inflammatory and anti-nociceptive effect, already known thanks to their historical usage, scientific studies have demonstrated antioxidant activity both in vitro and in vivo. Some evidence has highlighted the role of these nutraceuticals, mainly through their polyphenol and terpen content, in suppressing erythrocyte ROS production, lipid peroxidation, and decreasing oxidative DNA damage. The antioxidant effect, both local on joint cells and systemic on RBCs, in addition to the anti-inflammatory and anti-nociceptive action, make nutraceuticals valuable allies in OA treatment. Inherent in the many positive aspects to administer nutraceuticals, such as the safety and low side effects, it is required to highlight the limitations of their use to treat OA. The main limitation regards the oral bioavailability, when orally administered those molecules have to pass through the digestive system, thus undergoing several physicochemical changes, moreover, poor release from the extracted matrix, low solubility in the gastrointestinal fluids, low permeability through the intestinal epithelium [103]. Therefore, a very low amount of the nutraceutical components reach the joint; studies regarding the administration of glucosamine to treat OA, showed that the administration of 1.5 g/die of this molecule allowed to approximately 10 μM glucosamine to reach the joints [104]. The are several strategies to be followed to overcome these limitations, it can be mentioned the use of excipient molecules and of gastroprotection vehicles. Recently, the strategy to produce nutraceuticals in nanoparticle form is beginning to be explored. Nanoparticles reveal behaviors due to their small size and high volume-surface ratio suggesting interesting applications in the nutraceutical field [105].

**Figure 2:**
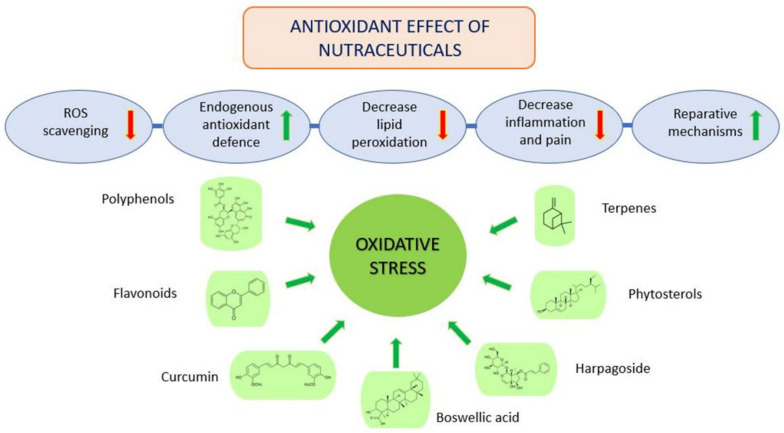
Oxidative stress protection diagram.

The future appears to hold much promise for nutraceutical antioxidants as a unique source of molecules with a safety profile and a vast multi-target potential in providing significant therapeutic benefits to patients affected by joint diseases (Figure 2).

## Data Availability

Not applicable.
